# Maternal Complications and Adverse Pregnancy Outcomes among Pregnant Women who Acquired Asymptomatic Bacteriuria in Addis Ababa, Ethiopia

**DOI:** 10.1155/2021/5254997

**Published:** 2021-08-13

**Authors:** Ketema Bizuwork Gebremedhin, Haile Alemayehu, Girmay Medhin, Wondwossen Amogne, Tadesse Eguale

**Affiliations:** ^1^College of Health Sciences, Addis Ababa University, Addis Ababa, Ethiopia; ^2^Aklilu Lemma Institute of Pathobiology, Addis Ababa University, Addis Ababa, Ethiopia

## Abstract

In this study, we aimed to document adverse pregnancy outcomes and maternal complications among pregnant women who acquired asymptomatic bacteriuria in Addis Ababa, Ethiopia. We used hospital-based prospective cohort study design in which we followed 44 pregnant women with asymptomatic bacteriuria confirmed by urine culture result of ≥10^5^cfu/ml of urine. We documented adverse pregnancy outcomes and maternal complications in terms of frequency, percentage, mean, and standard deviation. Additionally, we used Pearson's correlation coefficient to investigate associations of selected variables with perinatal death as one of adverse pregnancy outcomes. Of the 44 pregnant women enrolled in the study, complete data was collected from 43 participants with one lost to follow-up. Six (14%) of women developed fever and were treated with antibiotic during pregnancy, 26 (60.5%) delivered with cesarean section, two (4.3%) perinatal deaths within seven days of delivery, one miscarriage, and 4 (9.3%) newborns were found underweight. The mean birth weight of the newborns was 3.1 kg ± 0.60. Almost half 21(48.8%) were born before 37 weeks of gestational age. Fourteen (32.6%) of newborns were born asphyxiated. Twenty-two (51.2%) of newborns developed early neonatal fever within 48 hours of delivery and treated with antibiotic. Correlation coefficient analysis revealed that weight and gestational age of newborns at birth, Apgar score at 1^st^ and 5^th^ minutes of birth and miscarriage were positively correlated and significantly associated with perinatal death. The occurrence of unsought pregnancy outcomes were frequent, and substantial number of pregnant women developed maternal complications. Therefore, screening pregnant women for asymptomatic bacteriuria and treating may reduce the possible maternal complications and adverse pregnancy outcomes.

## 1. Introduction

Urinary tract infection (UTI) among pregnant women is the most widespread medical problem [1, 2]. UTI is among most common nosocomial infections [3] that may have significant contribution to the infection among pregnant women. Nosocomial infection would be acquired during either of their follow-up periods, some diagnostic procedures, and/or labor and delivery periods. The main reason that makes pregnant women more susceptible to the infections is pressure of gravid uterus to the bladder and immunologic and urinary tract vicissitudes [4–6] that occur due to physiological changes related to the pregnancy [6, 7]. Five decades ago, a study revealed prevalence of asymptomatic bacteriuria ranged from 2 to 10% of all pregnancies [8]; however, some recent studies showed prevalence of asymptomatic bacteriuria ranging from 2 to 15% [9, 10], and even some findings show higher rates of the infection [11–13]. UTI during pregnancy has been linked to increased risk of maternal and neonatal complications [14–17], and the complications are more severe when the women are comorbid with chronic inflammatory diseases, urinary tract abnormalities, autoimmune disorders, and other chronic illnesses [16, 18]. There are evidences that show that untreated urinary tract infection is the common cause of complications like premature rupture of membranes, low birth weight of infant, preterm birth, fetal intrauterine growth restriction, and postpartum endometritis [7, 16, 19, 20]. Moreover, previous studies reported that approximately 30-50% of pregnant women with confirmed pyelonephritis had preterm birth [21–23]. WHO estimated globally, major pregnancy-related complications including UTI accounts for 75% of maternal deaths, and many of them are preventable [24]. UTI and its complications are more intense in low- and middle-income countries (LMIC) [25]. This might be linked to scarcity of finance and logistics for timely screening of women for the infection [26]. At the same time, it is a less emphasized aspect towards pregnancy-related morbidity, mortality, and adverse pregnancy outcomes in this region [20]. In Ethiopia, there was no information that describes the effect of asymptomatic bacteriuria in women's health ailments and adverse pregnancy outcomes. However, Ethiopian mini demographic health survey 2019 [27] and other review article revealed [28] that infant mortality rated 77 deaths per 1,000 live births in 2005 which was decreased to 43 deaths per 1,000 live births in 2019 [27, 28]. Additionally, neonatal mortality decreased from 39 to 29 deaths per 1000 live births between 2005 and 2016 and remained steady since 2016 [27, 28]. Furthermore, a review of most predisposing factors of worst pregnancy outcomes showed that mean birth weight of babies born from 1990–2017 ranges from 2.077 to 3.147 kg [29]. Another review of literature in Ethiopia reported that pooled prevalence of preterm was 13.32% [30]. This shows that adverse pregnancy outcomes and maternal complications are still persisting health threats even if they are in declining order during the last decade [27]. In Ethiopia, to the best of our awareness, there is no study that explored adverse pregnancy outcomes and maternal complications related to asymptomatic bacteriuria. The aim of this study was to prospectively follow pregnant women with confirmed asymptomatic bacteriuria in Addis Ababa and explore the type and rate of occurrence of maternal complications and adverse pregnancy outcomes.

## 2. Materials and Methods

### 2.1. Study Context

The study was conducted in Addis Ababa, the capital city of Ethiopia, [31]. The city is found at above 2400 meters above sea level, 9°1′48 N 38°C 44′24′′E/9.03 degree N 38.7400 degrees east founded by Emperor Menelik at about 1892 [32]. The city has a projected 5,00600 population in 2021 [33]. The country's health delivery system follows the three-tier structure; the secondary and tertiary levels are comprised of general and specialized hospitals, and the coverage of each extends to larger portions of the population. A single tertiary level health care institution gives care for 3-5 million population while secondary level health care sector provides health care for 1-1.5 million population [34]. The city has over forty private hospitals and twelve governmental hospitals. We conducted this study in two governmental (Tikur Anbesa Specialized Hospital and Zewditu Memorial Hospital) and two private Mother and Child Health Care Specialty Centers (Hemen, and Anania Mother and Child Specialty Centers).

### 2.2. Study Design and Recruitment of Study Participants

The study design used was hospital-based observational prospective cohort of pregnant women that acquired asymptomatic bacteriuria and consented to be followed for adverse pregnancy outcomes and maternal complications. Initially, we cultured 281 urine of pregnant women with no symptoms of bacteriuria, no history of taking antibiotic two weeks before urine sample collection date, did not take large amount of water one hour before sample collection time, and not have any symptoms of sexually transmitted diseases before urine collection. Then, the forty four pregnant women with urine containing ≥10^5^cfu/ml of bacteria were considered as having asymptomatic bacteriuria.

### 2.3. Data Collection Instrument and Quality Control

The data collection tool was prepared from similar literatures and WHO guidelines. Five experts reviewed the quality of the questionnaire, and content validity was determined based on their reports. Accordingly, the content validity index was rated as 0.82, and appropriate modification was made and clarified for ambiguous items. The data collectors were experienced nurses and midwives working in the antenatal care centers of the study institutions.

### 2.4. Study Variables and Data Collection Techniques

The data collection techniques that we used were interview and reviewing the chart of the women enrolled in the study. The information was collected within three days of delivery of the followed women. Sociodemographic and maternal background characteristics like maternal age, marital status, educational status, occupation, monthly income per month, frequency of vaginal douching per day, sexual relation per week during this pregnancy period, (history of diabetes mellitus, hypertension, renal calculi, urinary tract infection before this pregnancy), (history gravidity, and parity), treatment for urinary tract infection in this pregnancy, and number of antenatal care visit in the current pregnancy were collected by interview. Maternal clinical characteristics like hemoglobin level before and after delivery, serology test status for HIV/AIDS, HipBSA, and VDRL test result for syphilis in the current pregnancy were reviewed from the chart of the women. Adverse pregnancy outcomes like perinatal death, miscarriage, (weight, length, gestational age at birth), (Apgar score at 1^st^ and 5^th^ minutes of delivery), respiratory distress syndrome and small for gestational age, and early neonatal infection requiring antibiotic within 48 hrs were reviewed from the chart of the women. Maternal complications like postpartum hemorrhage, (prenatal, intrapartum and postpartum period pyrexia requiring antibiotic), mode of delivery, premature rupture of membrane, induction of labor, symptomatic bacteriuria, pregnancy induced hypertension, eclampsia, preeclampsia, premature labor, endometritis, systemic infection, amnionitis, chorioamnionitis, pylonephritis, renal failure, cystitis, septicemia, hyperbilirubinemia requiring treatment, neonatal convulsion, hypoxic ischemic encephalopathy, neonatal encephalopathy, and severe neonatal morbidity were reviewed from the chart of the women.

### 2.5. Operational Definitions

Basic terms used in this study were operationalized as follows: maternal complications: complications that may occur during prenatal, labor, delivery, and postnatal period; pregnancy outcomes: results of conception and ensuing pregnancy; asymptomatic bacteriuria: presence of bacteria ≥ 10^5^cfu/ml in clean-voided midstream urine specimens of women without symptoms; symptomatic UTI: presence of bacteria greater than or equal to 10^5^cfu/ml) in clean-voided midstream urine specimens in a women with symptoms; large volume of water: consumption of greater than two glass of water one hour before reaching follow-up center; perinatal death: fetal deaths past 28 completed weeks of pregnancy plus the number of deaths among live-born children up to 7 completed days of life; preterm: fetus born before 37 weeks of gestation; small for gestational age: birth weights below the 10^th^ percentile for babies of the same gestational age; parity: number of delivery; gravidity: number of pregnancy; pregnancy-induced hypertension: new hypertension in a pregnant woman after 20 weeks' of gestation without the presence of protein in the urine; antenatal pyrexia requiring antibiotic: pyrexia treated with antibiotic during antenatal care period; intrapartum fever requiring antibiotic: fever requires antibiotic during labor and delivery period; postpartum fever requiring antibiotic: fever requires antibiotic during postnatal period; induction of labor: use of medications to induce labor; postpartum hemorrhage: excessive bleeding following the birth of a baby; and anemic: hemoglobin ≤ 12 g/dl.

### 2.6. Ethical Consideration

Ethical approval was obtained from the Institutional Review Board of Aklilu Lemma Institute of Pathobiology, Addis Ababa University (Ref.No.ALIPB-IRB/022/20112019) and from Addis Ababa Health Bureau (Ref. No. A/A/M/36975/227). Permission was obtained from the administration office of each of the study hospitals and heads of antenatal clinics; hereafter, the purpose of the study was clearly explained to the health care professionals working in the antenatal clinics. Verbal consent was obtained from each study participants after detailed information about the study was explained to them. They had also the right to withdraw and/refuse to participate in the study.

### 2.7. Data Analysis

Data was entered in to Epi-data version 3.1 and exported to SPSS version 22 statistical package software for cleaning, editing, and analysis. Descriptive statistics that include mean, standard deviation, frequency, and percentage were used to summarize the study variables. We used Pearson correlation coefficient to investigate association of selected variables with perinatal death as one measure of outcome. *P* value <0.05 was used to report findings as statistically significant.

## 3. Results

### 3.1. Sociodemographic and Background Characteristics

Among a total of forty-four pregnant women with acquired asymptomatic bacteriuria, 43 (97.7%) of them completed their follow-up (Table 1). The majority 27 (62.8%) were young in the age range of 25 to 34 years, and the mean age was 28 years (SD = 4.6). The majority 42 (97.7%) were married, sixteen (37.2%) had an experience of having two times sexual contact per week, and 31 (72.1%) had three times vaginal douching per day. Before the current pregnancy, 9 (20.9%) of the women had history of UTI, and 10 (23.3%) had renal calculi. More than half (51.2%; 22/43) of the pregnant women were anemic in their current pregnancy, their mean hemoglobin level before delivery was 12.4 (SD = 1.4), and their anemia level was reduced by 12% after delivery with a mean hemoglobin level of 12.5 ± 2.6. Nine (20.9%) of the women were living with HIV/AIDS and/or HipBSA, whereas only one woman was positive and treated for syphilis in the current pregnancy (Table 1).

### 3.2. Pregnancy Outcome and Maternal Complications

Among the whole pregnant women with ASBU followed for maternal complications and pregnancy outcomes; two (4.3%) perinatal death and one miscarriage were recorded. Four (9.3%) of newborns were found underweight while almost half 21 (48.8%) of newborns were born before 37 weeks of gestational age. Among a total of fourteen (32.6%) newborns asphyxiated, two of them (4.7%) had experienced severe asphyxia at 1^st^ minute; total asphyxia proportion found at 1^st^ minute was reduced to 7% in the 5^th^ minutes. Regarding maternal complication, seven (16.3%), six (14%), eight (18.6%), and five (11.3%) of the women experienced premature labor, preeclampsia, eclampsia, and pregnancy-induced hypertension, respectively. Five (11.6%) of the pregnant women who were with ASBU developed symptomatic bacteriuria later on during same pregnancy. Labors of 31 (72.1%) pregnant women were assisted by induction. The majority 26 (60.5%) of the women delivered by cesarean section (Table 2).

### 3.3. Association of Selected Variables with Perinatal Death

In order to measure association of variables with perinatal death, we used Pearson's correlation coefficient with *P* value <0.05 as a cut off point for significance level. There was no perfect correlation both positively and negatively with perinatal death. However, the positively associated variables: weight, gestational age at birth, Apgar score at first and fifth minutes, and miscarriage were found statistically significantly associated with perinatal death (*P* < 0.05) (Table 3).

## 4. Discussion

In this cohort of 43 pregnant women that acquired ASBU, even if no maternal death was recorded, various adverse pregnancy outcomes were documented. Some of the most prevalent adverse pregnancy outcomes noted were perinatal death, miscarriage, preterm birth, underweight, and asphyxia. Prevalence of perinatal death found in this study is higher by 1.3% than the global annual neonatal mortality rate [35], which is in line with trends of perinatal mortality in Ethiopia reported previously [36, 37]. This indicates that even if mortality of children under 14 years of age is in declining order globally during the last two decades [38], and is promising in Ethiopia [39], the increased perinatal death found by this study is bothersome. This could be due to uropathogens causing bacteriuria leading to adverse pregnancy outcomes through competing to nutrients, alteration of absorption of nutrients, and their metabolic mechanism [40–43] that would vanish immune functions of the women [43] resulting in fetomaternal complications [44]. Likewise, this study found four (9.3%) underweight, equated to be 90 low birth weight per 1000 births. This indicates high prevalence of low birth weight while the government and concerned stakeholders are endeavoring more to reduce by 30% until 2025 as targeted by WHO [45]. This finding is in line with the global prevalence of low birth weight (3-15%) [46] and Latin America (9%) [45] and Italy [41]. However, it is lower by 3.7% and 18.7% from sub-Saharan African and South Asian countries, respectively [45]. This pointed out a promising decline of low birth weight in Ethiopia compared to other regions. However, it is still high compared to global prevalence of low birth weight level [46]. This may be due to the newborns born to women who acquired asymptomatic bacteriuria during their pregnancy states. Therefore, bearing in mind, the consequences of low birth weight [47, 48] its haunting cause [36, 49, 50] including urinary tract infection [51], it is important to follow explicit intercession to reduce the prevalence of low birth weight in the country, which ultimately reduces prevalence of perinatal deaths [52, 53] and newborn-related complications [53, 54].

It is universal fact that preterm birth is fundamental causes of perinatal death [55], the global prevalence of preterm birth is 9.6% in 2005 [55], which still persists to 11% [56] in 2020. Here, this study found prevalence of preterm newborns born to women with ASBU while pregnant women were almost 4.4% times higher than the global estimated burden of preterm births [56]. In fact, finding of this study revealed lower prevalence of preterm birth when compared to report from sub-Saharan African and South Asian countries [57]. This indicates, even if finding of this study is promising compared to that of sub-Saharan and South Asian countries, it shows significant variation with the global burden of preterm birth burden. This may be due to the fact that preterm birth might be due to the complication resulting from ASBU during pregnancy [51]. Apgar score both at 1^st^ minute of delivery and 5^th^ minute has ground role in the survival rate and/or perinatal death of the newborns [58]. In this study, one out of three newborns was asphyxiated in their first minutes of birth, of which five percent of them were severely asphyxiated. Five minutes later, while almost all moderately asphyxiated newborns were relieved from their asphyxia, the severely asphyxiated newborns persisted with the asphyxia state found at birth. The two perinatal deaths reported in this study were those that had asphyxia that persisted after five minutes of delivery. This may perhaps be related to the precomorbidity of the women with other diseases while pregnant in addition to the known asymptomatic bacteriuria. This finding is two times higher than birth asphyxia revealed by study in the southern part of Ethiopia [59] and higher by 10.5% from study conducted in the northern part of Ethiopia [60], in line with another study from southern part of Ethiopia [61]. The higher prevalence of birth asphyxia found in this study may be due to the women being with ASBU and/or may be due to other comorbidities.

A significant number of pregnant women followed were found having delayed natural initiation of labor and required induction artificially. This may be due to the effect of comorbidity of the women with infectious diseases like symptomless bacteriuria that affects the endometrium.

Therefore, having in mind, the high rate of perinatal death and other maternal and newborn-related complications, it is essential to encourage and increase awareness of pregnant women at large the way to be healthy for both mother and fetus during prenatal, intrapartum and postnatal periods. At its instant point, first, pregnant women should increase the number of prenatal cares until delivery that helps the chance of identifying comorbidities, especially those with no symptoms like asymptomatic bacteriuria that may result in a number of adverse pregnancy outcomes as a hidden factor. Second, the health care professionals assigned in the prenatal care unit should always be up-to-date with recent updates of science in regards to their working areas and should try to identify pregnancy-related comorbidities and complications. Third, health care professionals assigned in the prenatal, perinatal, and postnatal care units should assess for each factor related to pregnancy complications in each visit of the pregnant women for care. The study has its own limitations, like small sample size that might not enable us to generalize to the wider population, and we recommend additional study with larger sample size and wide study area.

## 5. Conclusion

We documented numerous maternal and adverse pregnancy outcomes from the follow-up of pregnant women diagnosed with asymptomatic bacteriuria. The most common is perinatal death and immature labor which was supported by induction. Under weight, gestational age, Apgar score at first and fifth minutes, and miscarriage were found factors positively allied to the perinatal death. Therefore, there should be an intervention that handles the maternal complications and adverse pregnancy outcomes especially by focusing to the unseen comorbidities like asymptomatic bacteriuria. However, as the outcomes were recorded from only pregnant women with ASBU by not excluding other comorbidities, further follow-up study with the control group and larger sample size is recommended.

## Figures and Tables

**Table 1:** Sociodemographic, background, and clinical characteristics, *N* = 43.

Variable	Frequency (%)
Age in years	
15-24	10 (23.3)
25-34	27 (62.8)
35-49	6 (14)
Category of health care center attending	
Private	17 (39.5)
Government	26 (60.5)
Current marital status	
Married	42 (97.7)
Unmarried	1 (2.3)
Educational status	
Not read and write	1 (2.3)
Read and write	3 (7.3)
Primary school	6 (16)
Secondary school	9 (20.9)
College and above	24 (55.8)
Occupation	
Unemployed	31 (72.1)
Employed	12 (27.9)
Income per month	
≤100 $	14 (32.6)
>100 $	29 (67.4)
Frequency of vaginal douching per day	
≤2×	12 (28.0)
3×	31 (72.1)
History of DM before this pregnancy	
Yes	1 (2.3)
History of hypertension before this pregnancy	
Had	3 (7.0)
History of UTI before this pregnancy	
Had	9 (20.9)
Treated for UTI in this pregnancy	
Yes	13 (30.2)
Sero-status for HIV/AIDS and/or HipBSA during pregnancy	
Positive for HIV/AIDS	3 (7)
Positive for HipBSA	6 (14)
Status of VDRL test during pregnancy	
Positive for syphilis	1 (2.3)
Anemia status before delivery	
Anemic	22 (51.2)
Anemia status after delivery	
Anemic	17 (39.5)

**Table 2:** Pregnancy outcomes and maternal complications among pregnant women, *N* = 43.

Characteristics	Number (%)
Weight at birth	
<2.5 kg	4 (9.3)
2.5-4.00 kg	38 (88.4)
>4.00 kg	1 (2.3)
GA at birth	
<37 weeks	21 (48.8)
37-40 weeks	15 (37.9)
>40 weeks	7 (16.3)
Length at birth	
≤45	25 (58.1)
>45	18 (41.9)
Apgar score at 1 minute	
≤3	2 (4.7)
4-6	12 (27.9)
≥7	29 (67.4)
Apgar score at 5 minutes	
≤3	2 (4.7)
4-6	1 (2.3)
≥7	40 (93)
Small for gestational age	
Yes	5 (11.6)
Premature labor	
Yes	7 (16.3)
Preeclampsia	
Yes	6 (14)
Eclampsia	
Yes	8 (18.6)
Pregnancy induced hypertension	
Yes	5 (11.6)
Had symptomatic for UTI	
Yes	5 (11.6)
Miscarriage	
Yes	1 (2.3)
Incidence of antenatal pyrexia requiring antibiotic	
Yes	6 (14)
Incidence of prelabor rupture of membrane (PROM)	
Yes	26 (60.5)
Induced to initiate labor	
Yes	31 (72.1)
Mode of delivery	
SDV	16 (37.2)
OVD	1 (2.3)
CS	26 (60.5)
Intrapartum fever requiring antibiotic	
Yes	11 (25.6)
Postpartum fever requiring antibiotic	
Yes	27 (62.5)
Postpartum hemorrhage	
Yes	5 (11.6)
Respiratory distress syndrome	
Yes	2 (4.7)

**Table 3:** Association of selected variables with perinatal death under Pearson's correlation coefficient, *N* = 43.

Variables	Pearson's correlation coefficient	*P* value
Age in years	0.05	0.8
Category of health care center attending	0.3	0.08
Residence	0.07	0.7
Current marital status	0.03	0.8
Educational status	-0.2	0.3
Occupation	-0.1	0.5
Income per month	0.08	0.6
Frequency of vaginal douching per day	-0.1	0.4
Frequency of sexual relation per week	0.2	0.2
History of DM before this pregnancy	-0.03	0.03
History of hypertension before this pregnancy	-0.06	0.7
History of UTI before this pregnancy	0.2	0.3
Treated for UTI in this pregnancy	-0.1	0.4
Serostatus for HIV/AIDS and/or HipBSA during pregnancy	0.8	0.6
Status of VDRL test during pregnancy	-0.03	0.8
Anemia status before delivery	0.1	0.4
Anemia status after delivery	0.2	0.2
Weight at birth	0.4	0.02
GA at birth	0.5	0.01
Length at birth	0.3	0.06
Apgar score at 1^st^ minutes	0.6	0.01
Apgar score at 5^th^ minutes	0.5	0.01
Small for gestational age	-0.08	0.6
Premature labor	-0.1	0.5
Preeclampsia	-0.1	0.6
Eclampsia	0.2	0.3
Pregnancy-induced hypertension	-0.1	0.6
Had symptomatic for UTI	0.3	0.09
Miscarriage	0.7	0.01
Mode of delivery	0.05	0.7
PROM	-0.1	0.5
Induced to initiate labor	-0.1	0.5
Antenatal pyrexia requiring antibiotic	-0.1	0.6
Intrapartum fever requiring antibiotic	0.1	0.4
Postpartum fever requiring antibiotic	0.2	0.3
Postpartum hemorrhage	0.3	0.1
Respiratory distress syndrome	-0.05	0.8

## Data Availability

The data will be available from the corresponding author upon request.

## Abbreviations

Apgar: A: appearance, P: pulse rate, G: grimace, A: activity, R: respiration
ASBU: Asymptomatic Bacteriuria
CS: Cesarean section
DM: Diabetes mellitus,
G/DL: Gram per deciliter
GA: Gestation age
HiBSA: Hepatitis B surface antigen
HIV/AIDS: Human immunodeficiency virus/acquired immunodeficiency syndrome
IRB: Institutional review board
LMIC: Low- and middle-income countries
OVD: Operative vaginal delivery
PROM: Premature rupture of membrane
SVD: Spontaneous vaginal delivery
UTI: Urinary tract infection
VDRL: Venereal Disease Laboratory Research
WHO: World Health Organization.
